# Oleogels and Organogels: A Promising Tool for New Functionalities

**DOI:** 10.3390/gels8060349

**Published:** 2022-06-02

**Authors:** Miguel A. Cerqueira, Fabio Valoppi, Kunal Pal

**Affiliations:** 1 INL—International Iberian Nanotechnology Laboratory, Avenida Mestre José Veiga, 4715-330 Braga, Portugal; 2 Department of Food and Nutrition, University of Helsinki, P.O. Box 66 (Agnes Sjöbergin katu 2), FI-00014 Helsinki, Finland; fabio.valoppi@helsinki.fi; 3 Electronics Research Laboratory, Department of Physics, University of Helsinki, P.O. Box 64 (Gustaf Hällströmin katu 2), FI-00014 Helsinki, Finland; 4 Faculty of Agriculture and Forestry, Helsinki Institute of Sustainability Science, University of Helsinki, FI-00014 Helsinki, Finland; 5 Department of Biotechnology and Medical Engineering, National Institute of Technology, Rourkela 769008, India; kpal.nitrkl@gmail.com

Growing awareness concerning human health and sustainability has been continually driving the need to change consumers’ habits and develop new bio-based and environmentally friendly materials that could be used in new product formulations. One of the interesting materials that have emerged in the last 15 years are organogels and oleogels. They are semi-solid materials that contain a large fraction of organic solvents or liquid oil entrapped in a network of structuring molecules. These formulations appear as gels and solid-like structures that, according to the field of application, can be used to produce creams and mimic fats having a low amount of trans and saturated fatty acids. The increasing interest in these structures is related to the scientific and technological challenges, where researchers and companies have envisioned these structures to create new and innovative products. Some of the scientific challenges are related to: (i) the full understanding of gelator behaviour in different types of solvents, (ii) the lack of knowledge regarding the interaction and response of the gels to different constituents and processing conditions, and (iii) the unknown interaction and behaviour of bioactive compounds when added to organogels and oleogels.

Oleogels and organogels are hot topics, and while for oleogels the number of publications increased in the last 10 years from 13 to 151 (search in Scopus from 2011 to 2021 using the keyword “oleogel”), for organogels the number of publications has been quite constant increasing from 105 to 152 (search in Scopus from 2011 to 2021 using the keyword “organogel”). With this Special Issue, we bring some of the last findings related to the use of natural gelators and healthy oils in the development of oleogels/organogels and the delivery of bioactive compounds. We were able to present and discuss the most recent works on the development, production, characterization, and applications of oleogels and organogels. The published works are mostly focused on oleogels and food applications, but it is also possible to find good insights into the use of oleogels for pharmaceutical applications. There are also interesting research papers studying gelation mechanisms and the effect of different molecules on the final structure that could be used in different applications.

One of the exciting applications of oleogels is the delivery of functional molecules, where the incorporation of oil-soluble functional compounds can be explored. These can be performed not only at macroscale but also at micro- and nanoscale, resulting in different release behaviors and also different applications. The review of Pinto et al. presents and discuss the most recent works on the development, production, characterization, and applications of oleogels and other oleogel-based systems to deliver bioactive molecules in foods. The authors showed the main differences between hydrogels, oleogels, bigels, and emulgels and then focused on different methodologies for the preparation of oleogels and oleogel-based emulsions for food applications. In the end, they explored the use of those systems for the delivery of bioactive compounds in food applications [1]. In one of the research papers, Yilmaz and Demirci enriched thyme and cumin spices in oleogels based on sunflower wax and virgin olive oil. They showed that the incorporation could be successfully made without changing gel formation, stability, and gelation time, and consumer tests showed good approval by the consumers [2].

The prolonged consumption of high calorific and fatty (saturated/trans) products has shown to elevate the risk of metabolic syndromes such as obesity, coronary heart disease and diabetes. To mitigate the generally negative impact of lipid-rich products, several health organizations have called for improving the lipid profile of foods, that is, reducing saturated and eliminating trans fatty acids in foods (WHO, 2020) [3]. In addition to this, the food industry demands higher stability and better preservation of healthy food products in order to address consumers’ demands. Those demands can be answered by using tailor-made oleogels, which due to their unique viscoelastic, thermal, mechanical and optical properties, have been presented as good candidates. However, this goal is not always easy to achieve as developing oleogels for food applications presents several difficulties and challenges. In this regard, the work developed by Sahu et al. and Silva et al. studied the effect of natural waxes in the development of oleogels based on rice bran oil and olive oil, showing how oleogelation can be used in oil rich in monounsaturated and polyunsaturated fatty acids [4,5]. In addition to those works, Sahu et al. showed how different lecithins could be used to change the oleogel structure and crystallization process of candelilla wax [4]. In addition, Bharti et al. explored the effect of other molecules in the gelation of a natural wax. In this case, sunflower wax was used to gel sunflower oil and evaluate the effect of hydrophilic and hydrophobic surfactants [6].

Finally, in a research paper focused on pharmaceutical applications, Qureshi et al. studied the effect of graphene oxide on the development of oleogels based on cocoa butter and rice bran oil [7]. They used these oleogels for the delivery of ciprofloxacin hydrochloride in the in vitro drug release and ex vivo corneal permeation. Results showed that the prepared oleogels could be explored as potential delivery systems for ophthalmic applications.
